# Differentiation-dependent photodynamic therapy regulated by porphobilinogen deaminase in B16 melanoma

**DOI:** 10.1038/sj.bjc.6601760

**Published:** 2004-04-06

**Authors:** D Ickowicz Schwartz, Y Gozlan, L Greenbaum, T Babushkina, D J Katcoff, Z Malik

**Affiliations:** 1 Faculty of Life Science, Bar-Ilan University, Ramat-Gan 52900, Israel

**Keywords:** porphobilinogen deaminase, differentiation, melanoma, nuclear localisation, photodynamic therapy

## Abstract

Protoporphyrin IX (PpIX) synthesis by malignant cells is clinically exploited for photodiagnosis and photodynamic therapy following administration of 5-aminolevulinic acid (ALA). The expression and activity of the housekeeping porphobilinogen deaminase (PBGD) was correlated to PpIX synthesis in differentiating B16 melanoma cells. Differentiation was stimulated by two inducers, butyrate and hexamethylene bisacetamide (HMBA), both of which promote the formation of typical melanosomes and melanin, as well as morphological changeover. A marked decrease in total PBGD activity and PpIX synthesis was observed following stimulation by butyrate, while HMBA induced an opposite effect. In contrast, ferrochelatase levels remained unchanged. Photodynamic inactivation of the cells undergoing differentiation was largely dependent on the PpIX accumulation, which was modulated by the two inducers butyrate and HMBA. Fluorescence immunostaining with anti-PBGD antibodies revealed a major PBGD fraction in the nucleus and a minor fraction in the cytosol. This nuclear localisation pattern was confirmed by expression of PBGD fused to green fluorescence protein. We suggest that efficient photodynamic therapy of cancer facilitated by ALA administration can be enhanced using combined therapeutic modalities.

Photodynamic therapy (PDT) is used to treat both cancer and non-cancerous conditions. This treatment utilises a combination of photosensitising chemicals and visible light, usually light in the red or near infrared region, which is compatible with the absorption spectrum of the specific drug. (Dougherty *et al*, 1998).

Cancer cells *in vitro* and in solid tumours have been reported to exhibit an enhanced capacity for protoporphyrin IX (PpIX) synthesis facilitated by the administration of the pro-drug 5-aminolevulinic acid (ALA) (Zeitouni *et al*, 2003). This enhanced capacity is exploited clinically for PDT and photo-diagnosis (Peng *et al*, 1997). Aminolevulinic acid is the first intermediate molecule in the heme biosynthesis pathway and is formed when ALA synthase condenses glycine and succinyl CoA in the mitochondria. Two ALA molecules combine in the cytosol to form porphobilinogen (PBG). Porphobilinogen deaminase (PBGD) then combines four molecules of PBG into hydroxymethylbilane, which is turned into uroporphyrinogen III. Uroporphyrinogen III converts to coproporphyrinogen and then, after reuptake in mitochondria, to PpIX (Szeimies *et al*, 1996; Ponka, 1997; Gibson *et al*, 1998). Fluorescence microscopy data suggest that because of the localisation of PpIX in the mitochondria, the primary cause of cell death after PDT is mitochondrial phototoxicity (Iinuma *et al*, 1994).

Aminolevulinic acid is considered to be one of the most promising agents among the approved PDT sensitisers for a variety of malignant tumours, especially for skin cancers (Henderson *et al*, 1995; Abels *et al*, 1997). Malignant melanoma is the most life-threatening skin cancer and accounts for approximately three-quarters of all skin cancer deaths, since traditional forms of chemotherapy have little effect on this tumour (Rusciano, 2000; Bastuji-Garin and Diepgen, 2002). Aminolevulinic acid treatment of B16 melanoma cells induces PpIX biosynthesis and facilitates the photodynamic killing of these cells (Schoenfeld *et al*, 1994; De Rosa and Bentley, 2000; Juzenas *et al*, 2002). Several mechanisms have been suggested as being involved in the photodynamic process, including a lower intracellular iron concentration, lowered ferrochelatase activity, higher permeability of ALA into tumours, higher local temperature and higher PBGD activity in malignant cells (el-Sharabasy *et al*, 1992; Ackermann *et al*, 1998; Gibson *et al*, 1998).

Porphobilinogen deaminase is one of the rate-limiting enzymes in heme biosynthesis and it is expressed as two isoforms, a ‘houskeeping’ isoform present in all cell types and a second isoform expressed only in erythroid cells (Raich *et al*, 1986; Grandchamp *et al*, 1987; Sassa, 1990). We have recently shown that a major fraction of PBGD in C6 glioma cells is localised in the nucleus, in addition to the cytosolic fraction active in PpIX synthesis (Greenbaum *et al*, 2002). Furthermore, overexpression of the human housekeeping PBGD has been found to induce C6 differentiation toward astrocytes.

In this study, B16 melanoma cells were treated with two known differentiation agents, sodium butyrate and HMBA. Butyrate has been shown to accumulate during the G1 phase, influence gene expression and induce terminal differentiation (Toscani *et al*, 1988; Demary *et al*, 2001) even in melanoma cells (Nordenberg *et al*, 1987). It has been suggested that butyrate acts as a histone deacetylase inhibitor and thus the fraction of acetylated histones is dominant and the corresponding genes are transcribed. HMBA is a prototype of the hybrid polar differentiation inducer of many cell types, especially erythroleukaemia (Marks *et al*, 1987; Marks *et al*, 1994). HMBA induces the porphyrin biosynthesis pathway expression of globin genes and differentiation (Marks *et al*, 1987). Ortel *et al* (1998) have demonstrated that cellular differentiation increases the ability of a variety of cells to synthesise PpIX from exogenous ALA.

The purpose of this study was to correlate between ALA-PDT efficacy with modulation of PBGD expression and activity by chemically induced differentiation in B16 melanoma cells, and consequently to advance the understanding of the molecular basis of ALA-PDT specificity in clinical applications.

## MATERIALS AND METHODS

### Cell cultures

A B16 F10 mouse melanoma cell line was purchased from American Type Culture Collection. The cells were grown in RPMI 1640 (B16 F10) medium (Biological Industries, Beit-Haemek, Israel) supplemented with 10% foetal calf serum and antibiotics at 37°C in a humidified atmosphere with 5% CO_2_ and 95% air. The cells were passaged twice weekly.

### Cell differentiation induction

Hexamethylene bisacetamide (HMBA, 5 mM) or 2.5 mM sodium butyrate (Butyrate) (Sigma Chemical Co, St Louis, MO, USA) were added to the cells in complete medium. The cells were washed with PBS after 24, 48 or 72 h and harvested by centrifugation.

### 3-(4,5-dimethylthiazol-2-yl)-2,5-diphenyl tetrazolium bromide (MTT) assay

The effect on cell proliferation was measured using a modified MTT assay based on the ability of live cells to cleave the tetrazolium ring to a molecule that absorbs at 590 nm in active mitochondria (Mosmann, 1983). 2 × 10^3^ cells were grown in 96-well plates. The treatment medium was replaced and 20 *μ*l MTT reagent (5 mg/1 ml PBS) (Sigma, Chemical Co, St Louis, MO, USA) was added to each well. The cells were further incubated at 37°C for 2 h. For lysis of the cells, *N*,*N*-dimethyl formamide was added to the media for 5 h and absorbance was then measured at 570 nm.

### Scanning electron microscopy

The cells were fixed with 2.5% glutaraldehyde in phosphate buffer (pH 7.2), washed in the same buffer and post fixed with 2% OsO_4_. The third step of fixation was performed using a solution of tannic acid and guanidine hydrochloride. The triple-fixed cells were dehydrated in graded alcohol solutions. The alcohol was then exchanged for Freon-112 using graded Freon solutions. The cells were air-dried, gold coated and examined using a Jeol 840 scanning electron microscope.

### Transmission electron microscopy

The cells were fixed in 2.5% gluteraldehyde/2% formaldehyde in phosphate buffer solution (pH 7.4). The cells were removed with a Costarcell scraper, postfixed in 1% OsO_4_, dehydrated and embedded in Epon 812. Thin sections were prepared using an LKB Ultratome III and stained with uranyl acetate followed by lead citrate. The sections were examined using a JEOL 1200ex transmission electron microscope.

### Aminolevulinic acid treatment and photosensitisation

B16 melanoma cells were seeded into dishes and treated with butyrate and HMBA as described above. After 48 h, the medium was replaced in the dark by serum-free medium, with or without 0.1 mg ml^−1^ ALA (Clontech, Palo Alto, CA, USA) for 4 h. At the end of the incubation period, the cells were irradiated using a Vilber Lourmat lamp, VL-206BL, delivering a power density of 22.5 mW cm^−2^ at 360–410 nm (max. at 365 nm). Light intensity was measured with a Nova photometer (Ophir Optronics, Jerusalem).

### Annexin reaction

2 × 10^3^ cells were grown in 24-well plates, the cells were treated with ALA, butyrate and HMBA as described above and then irradiated for different light doses. After an additional 24 h, Annexin V-fluorescein isothiocyanate (FITC), propidium iodide (PI) and bisBENZIMIDE (Hoechst No 33258) were added to the cells according to the manufacturer's protocol (Sigma, Chemical Co, St Louis, MO, USA). Annexin allows fluorescent detection of Annexin V bound to apoptotic cells. The Annexin V is conjugated with FITC to label phosphatidylserine sites on the membrane surface. Propidium iodide was added to the cells in order to label the cellular DNA in necrotic cells where the cell membrane has been totally compromised. Hoechst was added in order to label the nucleus; a blue colour marked live cells, while a pink colour marked necrotic cells (Kuypers *et al*, 1996).

### DNA electrophoresis

Cells were treated with ALA, butyrate and HMBA as described above and then irradiated for different light doses. After an additional 24 h, the DNA was isolated and electrophoresed (Lund *et al*, 2002).

### Flow cytometry of cellular PpIX

B16 melanoma cells were grown on tissue culture plates and were incubated with or without 2.5 mM butyrate or 5 mM HMBA for 48 h. Aminolevulinic acid (Fluka, Sigma Diagnostics, St Louis, MO, USA) was diluted in RPMI-1640 medium to a stock solution of 10 mg ml^−1^ and a final concentration of 0.1 mg ml^−1^ was incubated with cells for 4 h. After incubation with ALA, the cells were washed with PBS (without Ca^+2^ and Mg^+2^) and scraped off with a rubber policeman. After 10 min of centrifugation at 1100 r.p.m., the medium was decanted and 0.5 ml of PBS (without Ca^+2^ and Mg^+2^) was added. The suspension was filtered and measured using a Fluorescence-Activated Cell Sorter (Becton Dickinson FACS Calibur, Mountain View, CA, USA). In all, 10 000 cells were measured in each sample (ex. 488 nm, em. LP 670 nm).

### PpIX accumulation assay

B16 melanoma cells were treated with butyrate and HMBA for certain periods of time. At 4 h before each point of time, 0.1 mg ml^−1^ 5-ALA (Clontech, Palo Alto, CA, USA) in serum-free medium was added to the cells. At the end of the incubation period, the cells were detached from the plates and centrifuged at 1100 r.p.m., followed by resuspension in glacial acetic acid. The cells were sonicated on ice and ethyl acetate was added (v v^−1^ 1 : 3). The samples were centrifuged at 3300 r.p.m. for 15 min, and the supernatant was transferred to 1 M HCl. After 5 min of shaking, the lower phase was collected and read using a spectrofluorometer (ex. 405, em. 620). The results were calculated as the mean of five experiments and expressed as %PpIX formed per mg protein during 4 h of incubation with 5-ALA.

### Western blotting

Proteins were quantified using the Bradford assay (Bio-Rad, Hercules, CA, USA) and resolved in a 12% polyacrylamide gel. The proteins were then transferred onto a nitrocellulose membrane using a semi-dry transfer apparatus (Bio-Rad, Hercules, CA, USA). After blocking of the membrane with 5% skim milk and 0.06% Tween-20 in PBS, the membrane was incubated with primary PBGD antibody (a generous gift from HemeBiotech, Sweden) primary ferrochelatase antibody (EnVirtue Biotechnologies, Walnut Creek, CA, USA), primary actin antibody (Sigma, Chemical Co, St Louis, MO, USA) or primary RCC1 antibody (Santa Cruz Biotechnology, California, USA) and goat anti-rabbit, goat anti-mouse and donkey anti-goat secondary antibodies (Jackson Immuno-Research, West Grove, PA, USA) in the same solution. Immunoreactive proteins were visualised with an enhanced chemiluminescence detection kit (Pierce Biotechnology, Rockford, IL, USA) used as recommended by the manufacturer.

### Porphobilinogen deaminase enzymatic activity assay

Porphobilinogen deaminase was assayed by determination of the absorbance of uropophyrin formed by light-induced oxidation of uroporphyrinogen, the immediate product of the enzymatic deamination. In all, 10^6^ B16 melanoma cells were detached from plates after incubation with the differentiation inducers and resuspended in PBS without Ca^2+^/Mg^2+^. After centrifugation, the pellet was resuspended in 1 ml 50 mM Tris (pH 8.2). In all, 250 *μ*l of the lysate was incubated with an equivalent amount of 50 mM Tris buffer, pH 8.2, containing 2% Triton X-100 and 100 *μ*l 0.5 mM PBG (Porphyrin Products, Logan, UT, USA) for 1 h with shaking at 37°C. The reaction was stopped with the addition of 10% TCA under exposure to ambient room light at room temperature for 10 min. After 10 min centrifugation (3300 r.p.m.), the supernatant was transferred to the spectrofluorometer (ex. 409 nm, em. 595 nm) (Spectronic Instruments, Leeds, UK). Porphobilinogen deaminase specific activity is expressed as pmol uroporphyrin formed (mg protein)^−1^ h^−1^.

### Nucleus-cytoplasm fractionation

B16 melanoma cells were harvested and resuspended in Tris-HCl buffer supplemented with EDTA, EGTA and the antiproteases leupeptin, aprotinine, PMSF and DTT (Sigma, St Louis, USA). For nucleus cytoplasm fractionation, the cells were homogenised on ice, followed by centrifugation at 2500 r.p.m. for 10 min (twice), and the nuclear fraction was resuspended in the above buffer. For Western blotting of each fraction, Triton X-100 was added and the sample was immersed in liquid nitrogen and thawed at 37°C intermittently, followed by centrifugation at 14 000 r.p.m. for 20 min. RCC1 levels were used as a nuclear fraction indicator (Figure 7).

### Porphobilinogen deaminase immunolabelling

Cells were seeded in eight-well chamber slides. After 24 h, the cells were fixed using 4% paraformaldehyde and were subsequently treated with 0.5% Triton X-100 for 30 min. Blocking was carried out with 6% skim milk, 3% BSA and 0.2% Tween 20 in 100% FCS. The cells were then exposed to the primary antibody, anti-PBGD, overnight at 4°C. Reacting rhodamine-conjugated donkey anti-rabbit (Jackson Immuno-Research, West Grove, PA, USA), ex. 570 nm, was visualised with fluorescence microscopy (Olympus AX70). As a control, anti-PBGD antibody was withdrawn from the samples and the cells were exposed only to the second antibody, rhodamine-conjugated donkey anti-rabbit.

### Preparation of fragments of PBGD fused to GFP

All the coding regions of the fragments were amplified from pHK-PBGD (pLY-81) (Greenbaum *et al*, 2002) using different primers for each fragment. The PCR products were cut by the restriction enzymes *Bgl*II and *Eco*RI, and were ligated to pEGFP-N1 (Clontech, Palo Alto, CA, USA) in frame. Constructs were verified by sequencing (Weitzmann Institute Biological Services, Rehovot, Israel).

### Transfection of plasmids to the cells

B16 melanoma cells were incubated in serum-free medium for 40 min, followed by 5 h incubation in a transfection solution containing 1.33 *μ*g ml^−1^ of each plasmid and 10 *μ*l lipofectamine reagent (Life Technologies, (GIBCO-BRL), Gaithersburg, MD, USA) in RPMI. Following transfection, the cells were transferred to a rich medium containing serum and antibiotic. GFP (ex. 395 nm) was visualised with fluorescence microscopy (Olympus AX70).

## RESULTS

**Figure 1:**
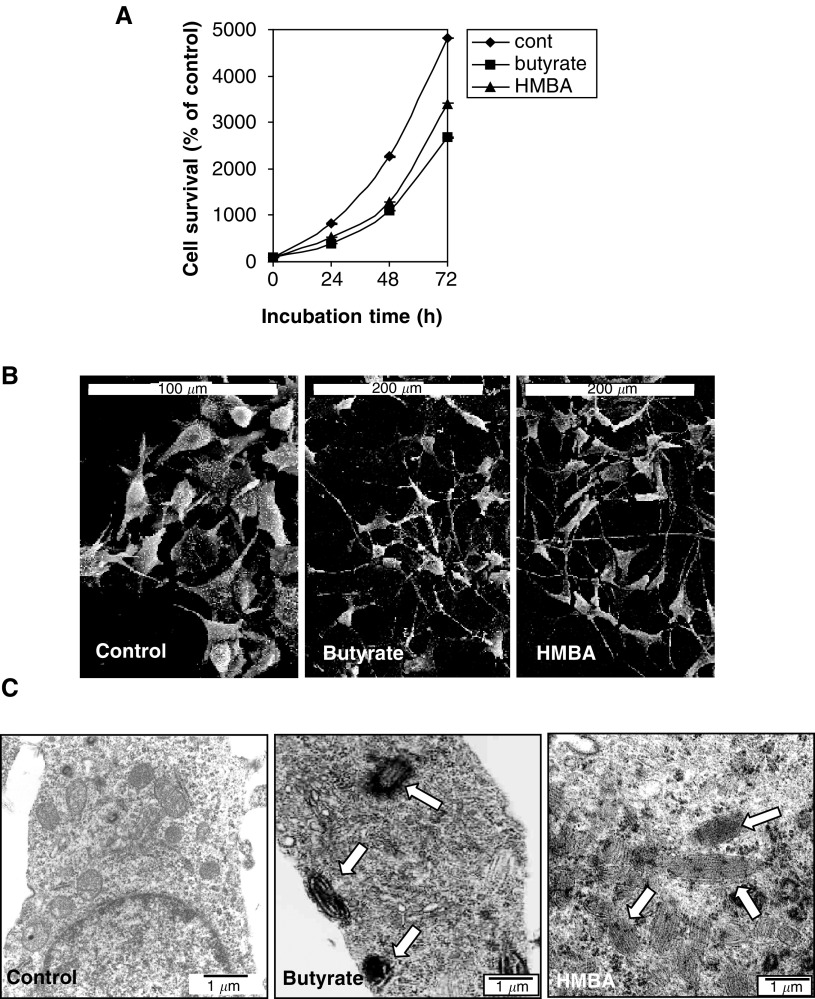
Effect of butyrate and HMBA on the morphology and cell proliferation of B16 melanoma cells. The cells were incubated with 2.5 mM butyrate or 5 mM HMBA for different times. (**A**) Presents the proliferation rate of the cells as detected by the MTT assay. Each point is the mean ± SE of two to three separate experiments. *P* value <0.005. (**B**) Scanning electron microscopy and (**C**) Transmission electron microscopy. The arrows indicate the presence of melanin.

Mitochondrial and cytosolic enzymes carry out heme synthesis, where PBGD, the third enzyme in the pathway, plays a major regulatory role. It has been suggested that the proliferation rate and cellular differentiation are major determinates in the regulation of heme pathway (Boldt, 1999). In order to characterise the interrelationship between PDT efficacy to porphyrin synthesis, PBGD expression and differentiation, we induced differentiation in B16 melanoma cells using two chemical inducers butyrate and HMBA. The attenuation in cell proliferation following butyrate and HMBA induction was assayed using the MTT reaction (Figure 1A

). The time-dependent reduction in cell viability by treatment with 2.5 mM butyrate or 5 mM HMBA after 24–72 h (Figure 1A), was accompanied by accumulation of 60% of the cells in the G1 phase, whereas for the cells that were not treated with a differentiation inducer 37% of the cells were in G1 (data not shown).

Melanin synthesis and formation of dendritic projections are considered as characteristic differentiation markers of B16 cells (Nordenberg *et al*, 1987), both of which were demonstrated by induction with butyrate and HMBA (Figure 1). The s.e.m. micrographs of cells after 48 h of butyrate and HMBA induction reveal a morphological changeover from round-shaped control cells to star-like flattened cells (Figure 1B). Transmission electron microscopy showed melanin synthesis, with formation and fusion of melanosomes following induction by both butyrate and HMBA in comparison to control cells (Figure 1C).

**Figure 2:**
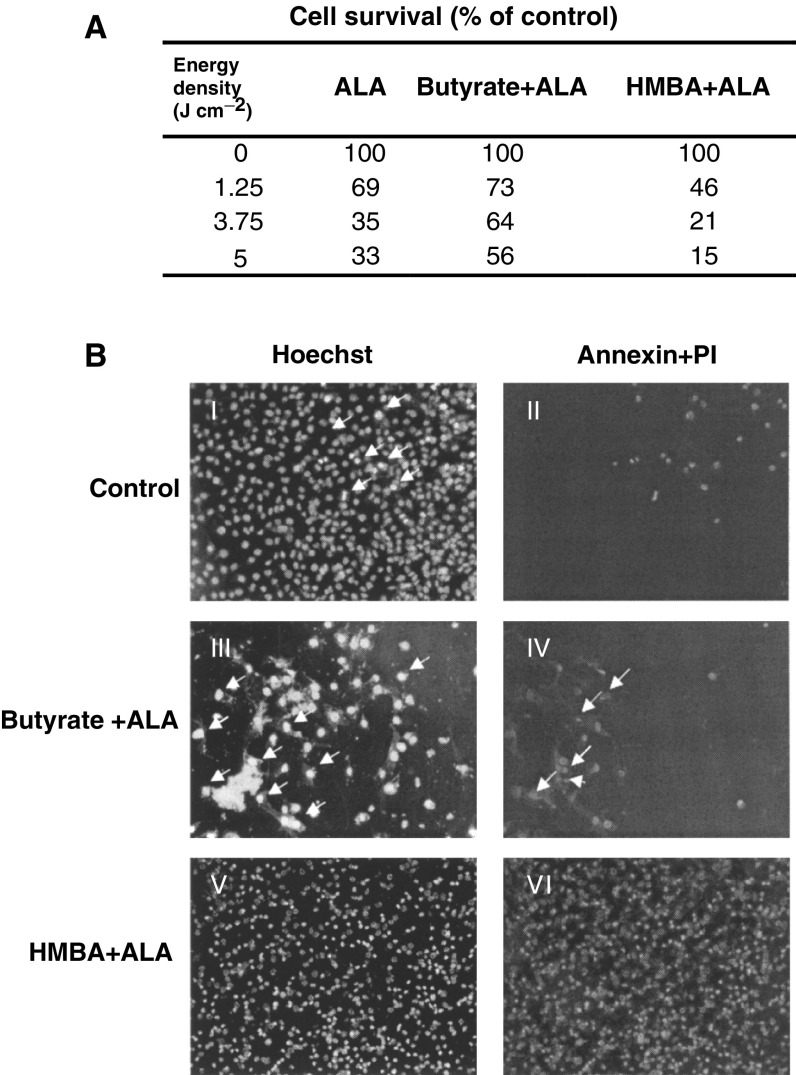
The effect of ALA and photo-irradiation on differentiated B16 melanoma cells. (**A**) The percentage of viable cells 24 h post photoirradiation was measured by an MTT assay. (**B**) Annexin reaction of irradiated cells with 3.75 J cm^−2^. The original photomicrographs mirror the intrinsic colors of the reactive materials I and II. Control cells were not treated with ALA. Arrows point to necrotic cells. Figs III and IV – cells were treated with butyrate +ALA. In III arrows point to necrotic cells, and in IV – the arrows point to apoptotic cells. Figs V and VI – cells were treated with HMBA+ALA. In V all the cells are necrotic.

**Figure 3:**
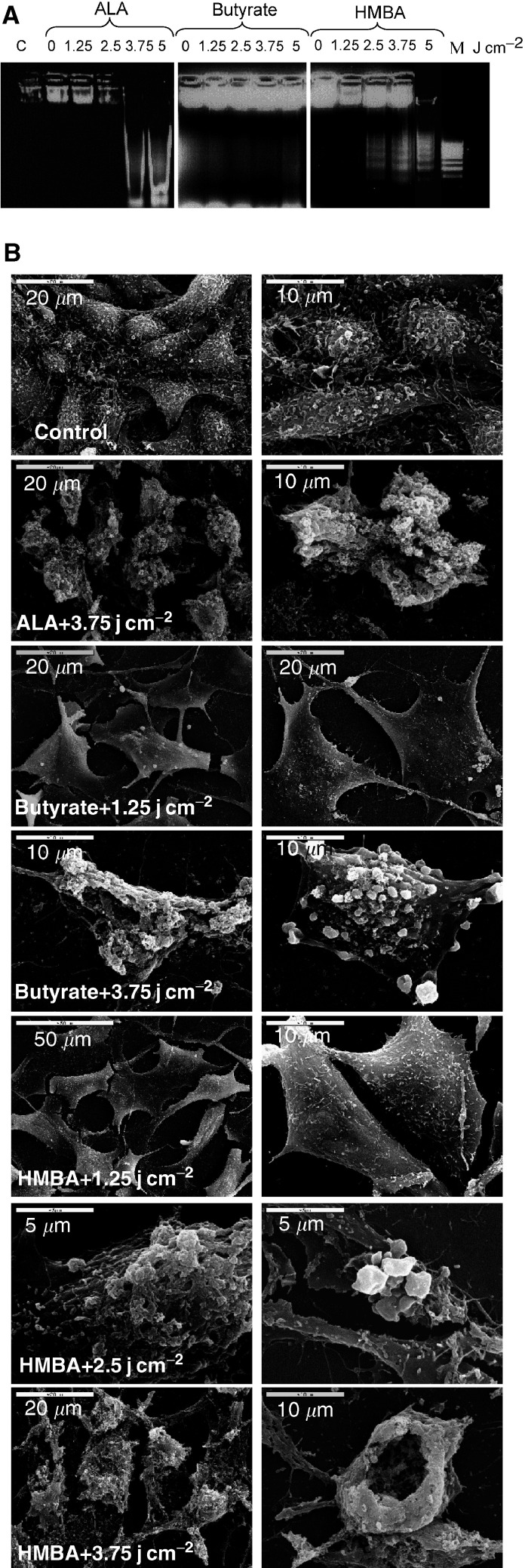
Effect of PDT on B16 melanoma cell death after different light doses (**A**) Electrophoretic ladder patterns: C-control cells, M-a marker for multiples of 200 bp fragments. (**B**) Scanning electron microscopy of cells following 5-ALA-PDT.

Photodynamic therapy efficacy when using butyrate or HMBA-induced cells treated with ALA showed a tight dependency between viable cells and specific differentiation route. The percentage of viable cells at 24 h post photoirradiation was lowest in HMBA-treated cells in comparison to ALA and butyrate-treated cells (Figure 2A

) measured by the MTT assay. The Annexin reaction of the photodynamic inactivated cells showed positive staining for most of the cells treated by HMBA+ALA, while this was true for only 30% of the butyrate+ALA-treated cells. The photo-inactivated HMBA+ALA cells revealed nuclear staining by PI, which is a strong indication of auto-membrane perforation due to PDT (Figure 2B). Cell death following photosensitisation was characterised by a typical DNA fragmentation pattern and necrotic and apoptotic features revealed with electron microscopy (Figure 3A

). Agarose DNA electrophoresis showed DNA laddering after ALA-PDT of the B16 cells and after HMBA-induced differentiation and ALA-PDT. No DNA laddering was revealed after butyrate-induced differentiation and ALA-PDT. Side by side, apoptotic and necrotic features such as the typical membrane blebbing and membrane rupture of heavily damaged cells were visualised by scanning electron microscopy (Figure 3B).

**Figure 4:**
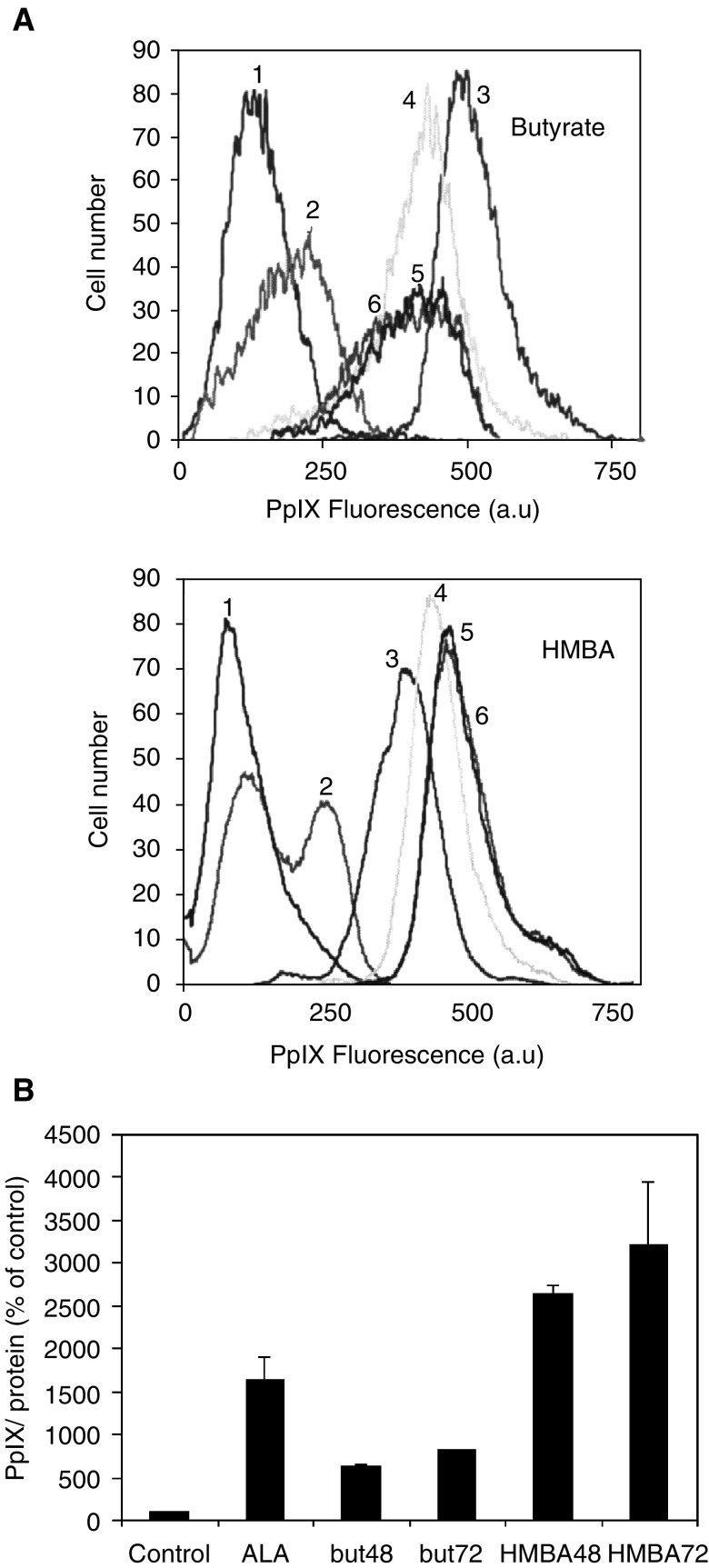
PpIX accumulation in B16 melanoma cells induced by butyrate and HMBA. (**A**) flow cytometry of cellular PpIX. 1. control 2. Butyrate or HMBA 3. ALA 4. 24h+ALA 5. 48h+ALA 6. 72h+ALA (a.u- arbitrary units) (**B**) PpIX accumulation measured by spectrofluorometery following extraction by acetic acid/ethyl acetate.

Hasan and co-workers have shown that induction of prostate cancer cell differentiation augments intracellular PpIX accumulation (Ortel *et al*, 1998). Similarly, DMSO induced differentiation of B16 cells enhanced by PpIX accumulation (Schoenfeld *et al*, 1994). Figure 4

shows that PpIX synthesis in the B16 cells is dependent on the differentiation agents, butyrate or HMBA, in two distinct and opposite modes. PpIX flow cytometry and PpIX extraction, both showed that butyrate induction plus ALA supplementation for 4 h promoted a low capacity of PpIX synthesis, in comparison to cells treated only with ALA. Alternatively, HMBA plus ALA induced a marked increase in PpIX synthesis and accumulation in the B16 cells (Figure 4).

**Figure 5:**
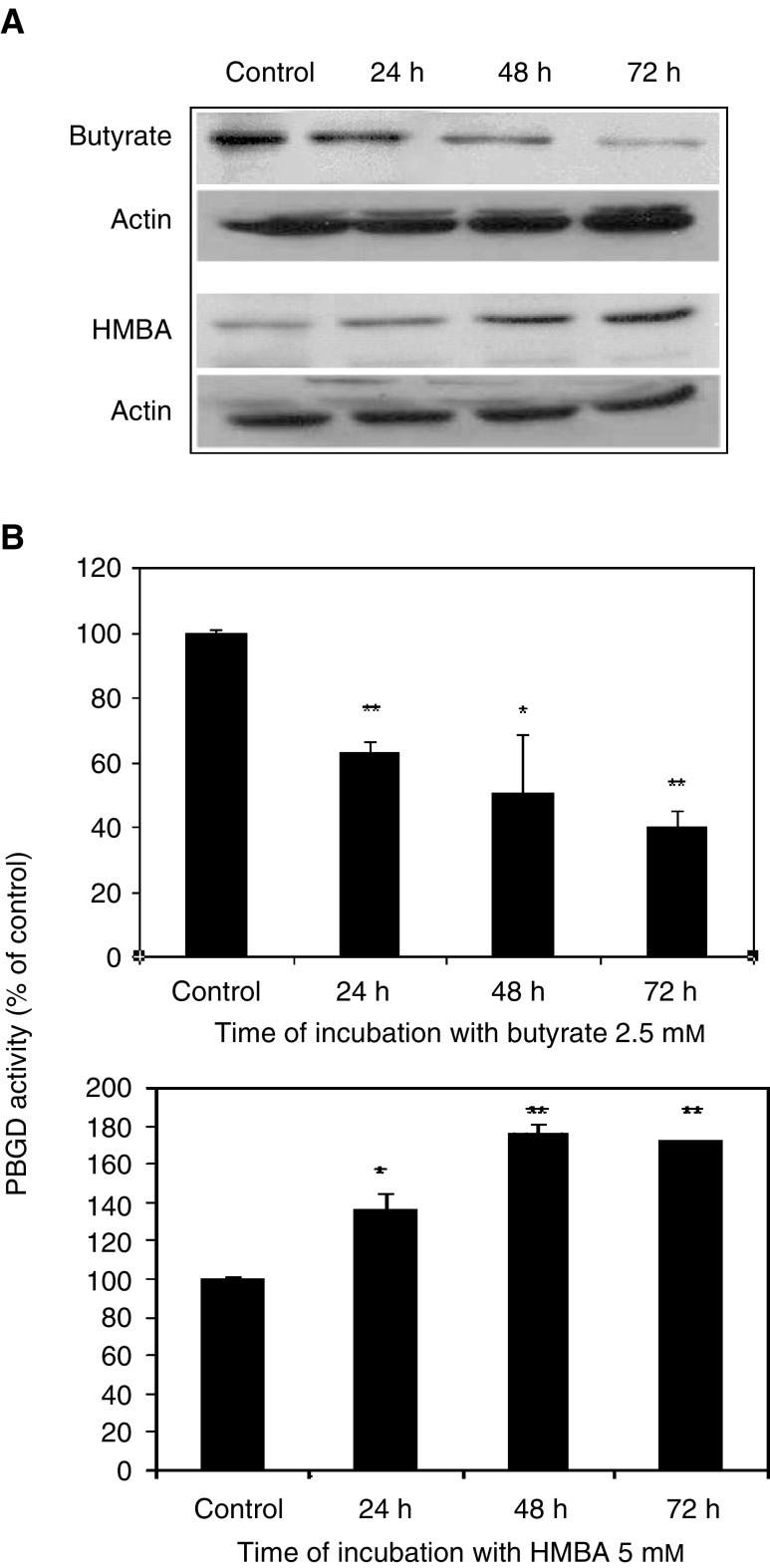
Effect of butyrate and HMBA on total enzymatic activity of PBGD in B16 melanoma cells. (**A**) Western blot analysis followed by chemiluminescence detection of cells that were treated with 2.5 mM butyrate or 5 mM HMBA for different times. The data presented here are from a representative experiment repeated three times with similar results. (**B**) Time response curve of the effect of 2.5 mM butyrate and 5 mM HMBA on PBGD activity. Each point is the mean±s.e. of two to three separate experiments, which were performed in triplicate. ^*^P<0.05, ^**^P<0.005.

It has been suggested that during differentiation and reduction in heme synthesis, the activity of the third enzyme in the porphyrin synthesis pathway, PBGD, would be altered accordingly (Ortel *et al*, 1998). Porphobilinogen deaminase during butyrate-induced differentiation of B16 cells was analysed by Western blotting, revealing a time-dependent decrease in total PBGD protein (Figure 5A

). The specific enzymatic activity of PBGD was reduced (Figure 5B) as a time-dependent effect following butyrate treatment. In contrast, the effect of the second inducer, HMBA, was the opposite, revealing a time-dependent increase in total PBGD (Figure 5A), as well as an increase in the specific enzymatic activity (Figure 5B). Actin levels were used as an internal protein control.

**Figure 6:**
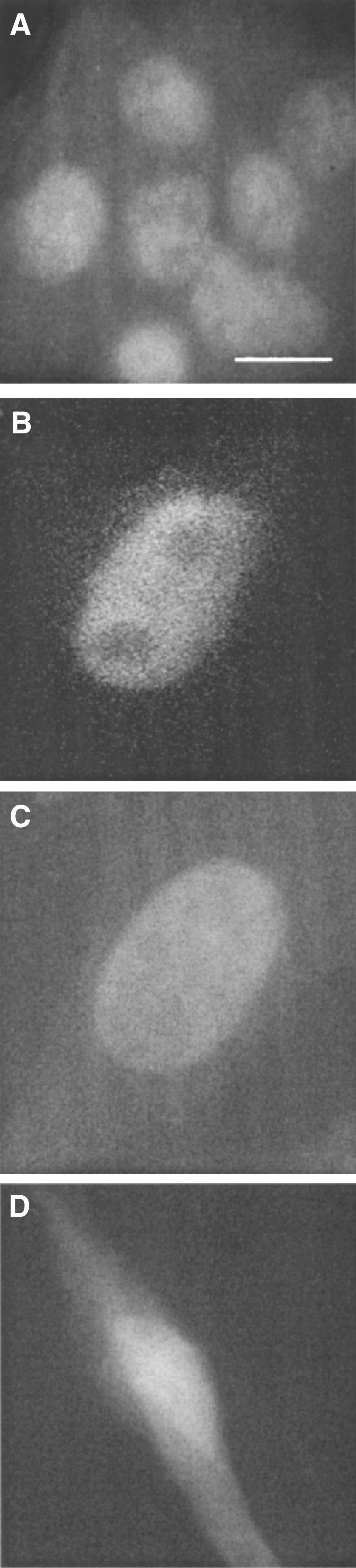
Nuclear localisation of PBGD in B16 melanoma cells (**A**) In immuno-labelling, the nuclear versus cytoplasmic distribution of PBGD was similar for all experiments. In control cells, in which anti-PBGD antibody was withdrawn from the samples, there was no signal. Original magnification X 60, bar 5 *μ*m. (**B,C**) Over expression of human housekeeping PBGD fused to GFP on the N-terminal (B) or the C-terminal (C). (**D**) The negative control for nuclear GFP localisation was cells expressing the wild type GFP plasmid and revealing diffused fluorescence. Original magnification X 100, bar 5 *μ*m.

We have already shown that the major fraction of PBGD in C6 glioma cells was found in the nucleus (Greenbaum *et al*, 2002). Figure 6

reveals the subcellular localisation of PBGD in B16 melanoma cells. The fluorescence immunolabelling with specific anti-human PBGD antibodies shows that a large fraction of PBGD was localised in the nucleus of the B16 melanoma cells (Figure 6A). This result was affirmed by transfection of the B16 cells by the plasmids pLY1 and pLY2, encoding PBGD fused to GFP at either the C terminal or N terminal, respectively. Expression of pLY1 and pLY2 in the B16 cells showed clearly a major fraction of newly synthesised fused GFP-PBGD in the nucleus (Figure 6B–C), while control cells transfected with wild-type GFP (pE-GFP-C1) showed diffused fluorescence throughout the cytoplasm (Figure 6D).

**Figure 7:**
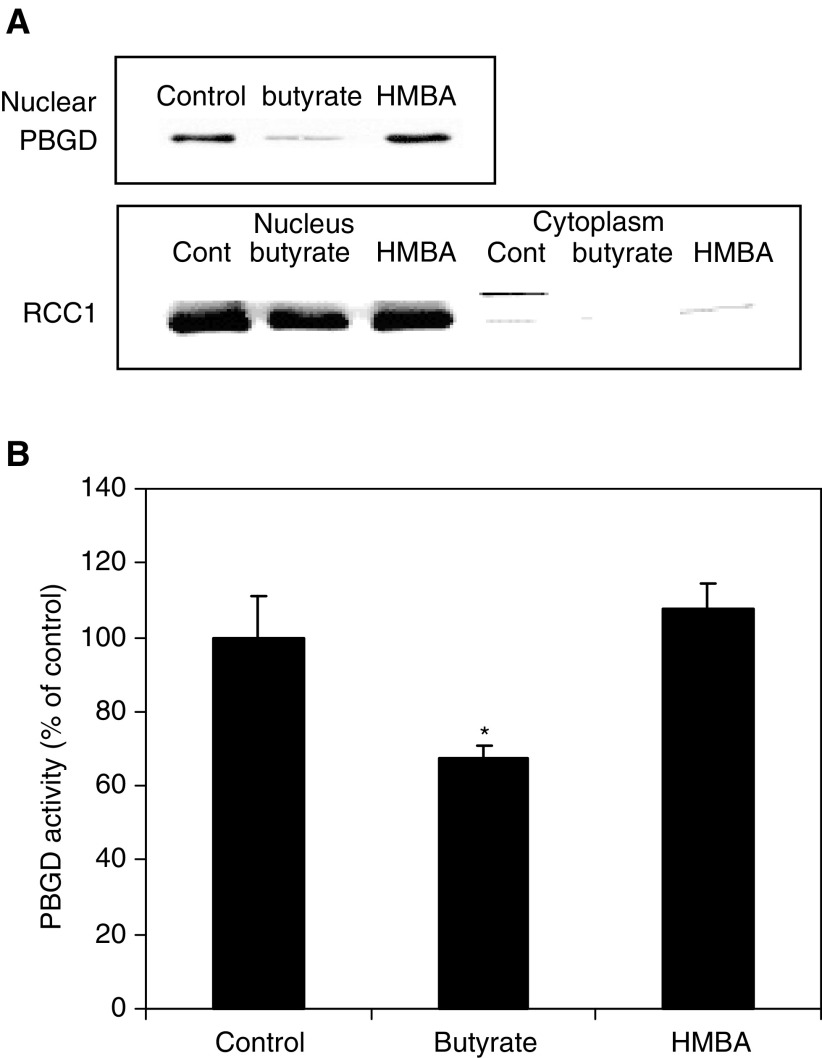
Effect of butyrate and HMBA for 48 h on nucleus PBGD fraction. (**A**) Western blot analysis using rabbit anti-PBGD; RCC1 is a specific nuclear marker detected by rabbit anti RCC1. (**B**) The enzymatic activity of nuclear PBGD (^*^P<0.05).

**Figure 8:**
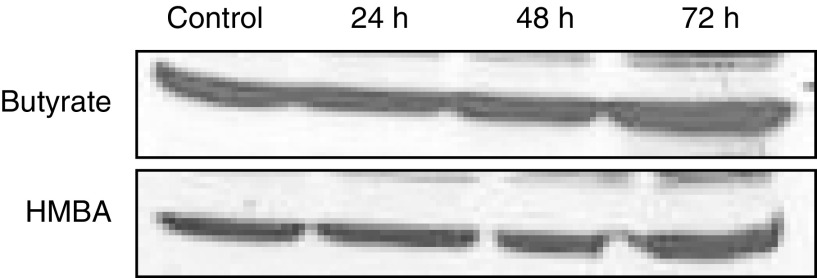
Effect of differentiation on protein expression of ferrochelatase in B16 melanoma cells. Cells were treated with 2.5 mM butyrate or 5 mM HMBA for different times. The data shown here are from a representative experiment repeated three times with similar results.

Western blotting and specific enzymatic activity of the nuclear fraction of PBGD during induced differentiation revealed a time-dependent decrease in butyrate-treated cells. On the other hand, HMBA-treated cells showed an opposite effect: nuclear PBGD protein levels and activity were increased in a time-dependent manner (Figure 7A–B

). Ferrochelatase, another key enzyme in heme biosynthesis, was analysed by Western blotting and showed an unchanged enzyme content during B16 differentiation induced by HMBA or butyrate (Figure 8

).

## DISCUSSION

Rapidly proliferating cells manifest an accelerated capacity for PpIX synthesis following ALA treatment. This property is widely used for PDT and photodiagnosis of solid tumours. The relationship between heme biosynthesis and proliferation, differentiation and apoptosis is not well understood. It is conceivable that PBGD is a rate-limiting enzyme and a regulatory checkpoint in porphyrin biosynthesis. It has been proposed that changes in the PBGD activity are closely related to growth (Boldt, 1999). Furthermore, PBGD and ALA dehydrase (ALAD) activities seem to be greater in some malignant tissues than in normal tissue (Mamet *et al*, 1994). Our results revealed that total PBGD was dramatically affected during differentiation of B16 melanoma cells. Stimulation with butyrate induced melanocytic differentiation accompanied by a reduction in cellular PBGD as determined by Western blot analysis, while HMBA induced similar differentiation markers with a prominent increase in total PBGD. Similarly, the enzymatic activity of PBGD was well correlated with total PBGD content following butyrate and HMBA treatment. No changes were found in total ferrochelatase during butyrate and HMBA stimulation in Western blot analysis. It has been previously shown that malignant tissues contain low levels of ferrochelatase, which explains the high accumulation of PpIX following ALA treatment (Ponka, 1997). Thus, PBGD seems to be the main checkpoint in PpIX synthesis in our B16 melanoma system.

Our present independent methods of immunolabelling and GFP-tagging reveal dual localisations of PBGD in B16 melanoma cells, one cytosolic and the other nuclear. Recently, we have shown interaction between PBGD and a nuclear protein Ran-binding protein (Greenbaum *et al*, 2003). These findings are indicative of a possible dual function of PBGD, one enzymatic in the cytoplasmic heme pathway and an unknown role in the nucleus. Tagging the GFP to either the N or C terminal of PBGD exhibited the same nuclear localisation pattern, although the specific localising sequence is not yet defined.

Recently, we have demonstrated that during mitosis chromatids were intensely stained for PBGD in comparison to the interphase chromatin (Greenbaum *et al*, 2003 b). Using the yeast two-hybrid system, the RanBPM, the nuclear Ran-binding protein, was shown to interact with PBGD. The molecular function of PBGD-Ran BPM in the nucleus is now being explored, having in mind the finding that PBGD is bound to chromosomes. Similarly, RanBPM was both localised in the nucleus and cytosol, and especially surrounding the centrosome (Nishitani *et al*, 2001). We speculate that the unknown nuclear role of the PBGD in addition to its cytosolic enzymatic activity required for heme synthesis is probably interrelated to cell transformation and differentiation.

The prominent PBGD activity in tumours, which is responsible for the enhanced porphyrin synthesis following ALA treatment, is exploited clinically. Malignant glioma (Schoenfeld *et al*, 1988), oral cavity cancer, colonic and gastrointestinal dysplasia (Stummer *et al*, 1998a), peritoneal endometriosis (Stepp *et al*, 1998), lower urinary tract tumours (Hillemanns *et al*, 2000), bladder cancer (Kriegmair *et al*, 1999), malignant mucosa in head and neck cancer (Kriegmair *et al*, 1996) and basal and squamous cell carcinoma (Betz *et al*, 1999), all demonstrate eminent PpIX synthesis and specific accumulation. Our results show a direct correlation between total PBGD (nuclear and cellular), its enzymatic activity and PpIX accumulation. The reduced cellular PBGD following butyrate induction resulted in a proportional decrease in the PpIX synthesised from exogenous ALA, while HMBA treatment induced a prominent but opposite effect of increased cellular PBGD and PpIX production.

From the present results and from earlier studies, two patterns of differentiation can be outlined, one associated with upregulation and the other with downregulation of PBGD expression. Ortel *et al* (1998) have shown that induction of keratinocyte differentiation augments intracellular PpIX accumulation. Similarly, DMSO was found to induce differentiation of B16 melanoma cells and enhance PpIX accumulation (Schoenfeld *et al*, 1994) and Mamet *et al* (1994) demonstrated increased activity of PBGD in malignant cell lines. Interestingly, higher heme biosynthetic enzyme activities such as PBGD and lower PpIX precursor concentrations were found in Barrett's oesophagus and adenocarcinoma of the oesophagus (Betz *et al*, 1999).

Oleinick *et al* (2002) concluded that cell death following PDT is in many cases due to a combination of apoptosis and necrosis. Furthermore, even for a specific photosensitiser used for PDT, it is difficult to determine the unique cellular response. Our results demonstrate that ALA-PDT of B16 melanoma cells results in a combined effect of apoptosis and necrosis, as revealed by a variety of manifestations, including ALA-PDT-induced blebbing, DNA laddering, membrane rupture, mitochondrial inhibition and ultra-structural damage. The extent of cell death during ALA-PDT is shown to be closely related to the PBGD activity and PpIX accumulation, while the mechanism of cell death cannot be predicted during this treatment. We may conclude that the ALA-PDT outcome largely depends on PBGD cellular levels, which in turn may change with specific up and down regulations during tumour differentiation pathways.
